# Supplementary figures and images for: Comprehensive analysis of locomotion dynamics in the protochordate Ciona intestinalis reveals how neuromodulators flexibly shape its behavioral repertoire
Source: PLoS Biol. 2022 Aug 4;20(8):e3001744. doi: 10.1371/journal.pbio.3001744 (PMC9352054; doi:10.1371/journal.pbio.3001744)

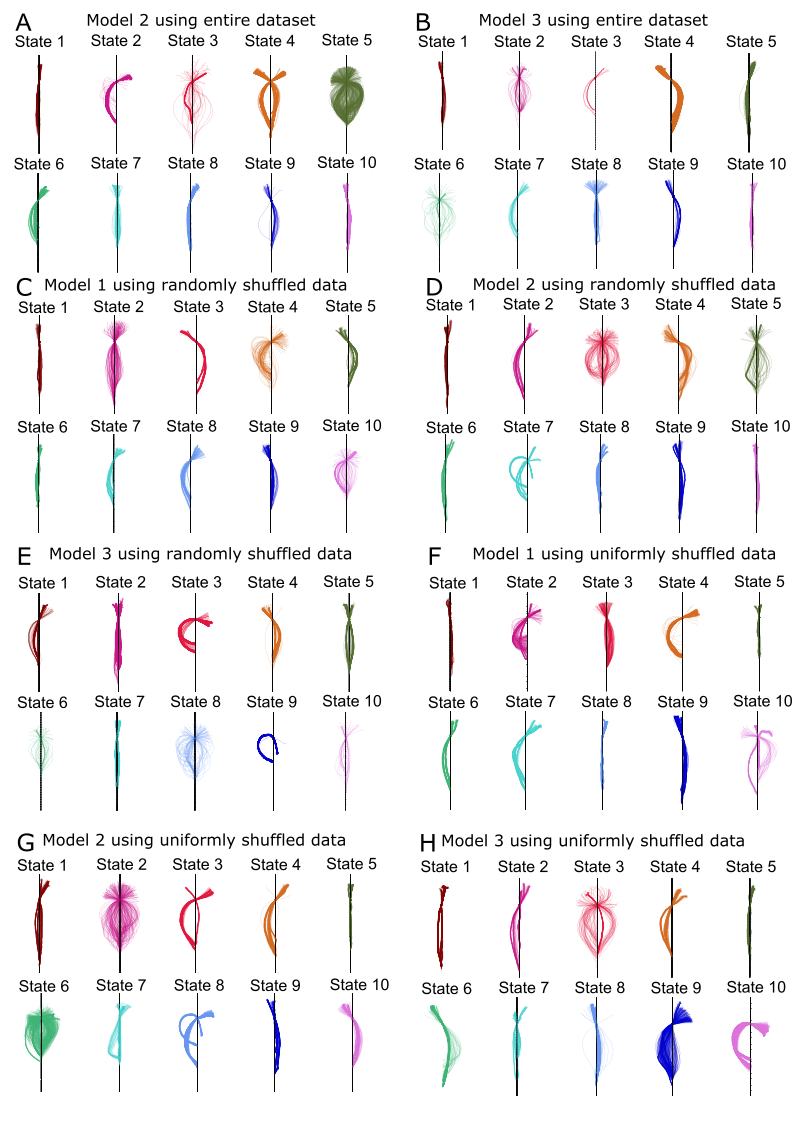

Supplement: S17 Fig — Postures/skeletons were randomly sampled from the training dataset for each of the 10 different HMM states, aligned such that the neck points coincide and are collinear with tail-ends on a vertical line for each of the additional HMM models trained. (A, B) Skeletons sampled for the 10 states as learned by the 2 additional models trained on the entire dataset. (C–E) Skeletons sampled for the 10 states as learned by the 3 models trained on the 3 randomly split subsets. (F–H) Skeletons sampled for the 10 states as learned by the 3 models trained on the 3 uniformly split subsets. Ciona skeletons were randomly sampled from our dataset that can be found in https://doi.org/10.5281/zenodo.6761771. (TIF) [file pbio.3001744.s017.tif]
