# Supplementary material for: Comprehensive analysis of locomotion dynamics in the protochordate Ciona intestinalis reveals how neuromodulators flexibly shape its behavioral repertoire
Source: PLoS Biol. 2022 Aug 4;20(8):e3001744. doi: 10.1371/journal.pbio.3001744 (PMC9352054; doi:10.1371/journal.pbio.3001744)
Supplement: Attachment — Submitted filename: Response to reviewers.docx [file pbio.3001744.s102.docx]

REVIEWERS' COMMENTS:


Reviewer #1:

I enjoyed reading and reviewing this paper. This project fills the need for more comparative, quantitative studies of neurobehavior beyond the most popular model organisms. The computational ethology is performed using up-to-date techniques and covers the behavioral space comprehensively. While the paper may read more like a PLOS Computational paper than a PLOS Biology paper, I think the broader readership of PLOS Biol. would appreciate this work. It may for many researchers be a model example for their own attempts to quantify and publish behavior outside of the mainstream model organisms.

Note that I am not an expert in C. interstinalis or very familiar with many of the pharmacological agents used in this study.
We thank the reviewer for their kind comments. Like the reviewer we also hope that our work will encourage others working with non-mainstream model organisms to explore and quantify behavior.

Comments:

1) I would like some more detail on the experiment. How were the animals prepped? I didn't see a reference to any "standard methods." Were recordings made immediately after preparing samples or were they left for some incubation time? How long did the sessions last? Were controls randomized across the data collection or did the wild-type (control) collection occur separately from the pharmacological experiments? In general when "control" is used I would like a clarification if this was a control for the specific type of experiment or was this just the wild-type data presented earlier.
We thank the reviewer for raising these important points regarding methodology. We have substantially extended the following methods subsections: “Egg fertilization, embryo and larval rearing conditions”; “Experimental setup” and we have added a new section titled “Experimental procedure”. We now address how the animals were prepped including referencing standard methods as well as any deviations from these methods. In the Experimental procedure section, we now indicate that controls for all drugs corresponded to wild-type data apart from Dopamine for which the appropriate control were 64 animals treated with ascorbic acid in Artificial Sea Water (ASW).

We also now clarify in the methods that controls were randomized across the data collection. In fact, every video acquisition round, included the acquisition of at least one wild-type animal (or ASW+ Ascorbic acid when we were collecting the Dopamine treated animal data).

We would like to note that following the reviewer’s advice we have substituted the word “control” with “wild-type” in the main text, figure legends and supplementary Information files. When dopamine is presented in the data we clarify in the main text and figure legends that the corresponding control is ASW+ Ascorbic acid.

Re: the raw data and the imaging. I may have missed this, but what are the dimensions of the larvae and the dimensions of the container? The swimming is constrained to 2D I guess, but how? Is this due to a physical or behavioral barrier (e.g. liquid height)? How are the larvae illuminated for imaging (e.g. ring light?).
We thank the reviewer for pointing out these omissions in our manuscript. In the Figure legend of Fig 1B and in the methods section “Egg fertilization, embryo, and larval rearing conditions”, we indicate the average length of the Ciona larvae used in our experiments (1330.61μm or equivalently115.10 pixels).

In the revised Figure legend of Fig1 and in the “Experimental setup” methods section, we indicate the diameter (10mm) and depth of the arena (3mm), as well as the volume of liquid it can hold (240mm^3^). In the revised “Experimental setup” section we now explain that the animals are not constrained to 2D but the relatively shallow arena means that the 3^rd^ dimension available to the larvae is rather limited.

The reviewer is correct in thinking that we used an illumination ring (with IR LEDs). We have extensively expanded the “Experimental setup” section to describe in detail the behavioral setup including providing details on how the larvae were illuminated.

2) Fig.1. I don't think it is at all useful to show the GUI of the software used to capture and process images. It would be much more informative to get a very good schematic or image of the set up and a very good image of the raw data (i.e. larvae before processing).
We thank the reviewer for this suggestion. Following their advice, we have replaced the GUI snapshots with a schematic of the setup (Fig 1A) and a single frame of the raw data before it is processed in Tierpsy (Fig 1B). In addition, we have generated a supplemental figure (new Supplemental Figure 1) which has multiple pictures of the setup and 3D renderings of various 3D printed parts that we have used to build our setups. We have also included in the methods a link to our GitHub repository where further information (pictures, schematics of the electronics, STL files for the 3D printed parts etc) can be found. Finally, we have extensively expanded the “Experimental setup” section in the methods to provide more details on the setup.

3) The authors state that "given our assumption that the head segment is rigid." Can't this be shown rather than assumed?
We agree with the reviewer that experimentally demonstrating the rigidity of the head segment would be better than just assuming that this is the case. We have therefore we have performed a series of analyses to show that the head segment is rigid. We performed two types of analysis. Firstly, we examined the amount by which the head segment deviates from a straight line in each case. To estimate this, we calculated the perpendicular distance of each of the skeleton points from a straight line drawn between the head tip point and the neck point. For a comparison with the tail segment, we then estimated the deviations of the tail from a straight line. For the tail segment, we calculated in a similar fashion, the distances of the skeleton points from a line drawn between the neck point and the tail tip. We have provided a supporting schematic and bar plots for the measured deviations in the new figure Fig. S2. As seen from the bar plots, the deviations from a straight line in the head region is very minimal when compared to the tail region.

In addition, we have now provided some plots of curvatures along the 49 points from three randomly sampled video clips in the dataset which also indicate that the head region exhibits lower curvature values, with a much narrower range as compared to the tail region. These plots are also now provided in the new Fig. S2. The analyses are also explained in the modified text within the methods section under the subsection titled “Head rigidity determination”.

4) Fig 3B. It's not clear what is being plotted here. Is it a specific skeleton segment? Center of mass?
 We agree with the reviewer that this was not clearly explained in the figure legend. What we are plotting are Ciona larva skeletons generated by Tierpsy and this is now stated in the figure legend of Figure 3 for panels B and C.

5) re: light stimuli. As a reader not familiar with this organism, I wonder what the purpose was to use red, green, and blue light in addition to white light.

We thank the reviewer for raising this point. Previous work by Nakagawa et al (1999)(1) determined the action spectrum of larval motility (amongst other experiments they measured larval speed across different wavelengths of light). This study showed that the strength of the light-off response was dependent on the wavelength of light that was used. In our study we wanted to determine whether we could distinguish further differences in swimming activity and behavioral actions that different wavelength light-off stimuli may elicit. This is now explained in the modified text within the results section under the subsection titled: “Light stimuli modulate postural dynamics and behavioral space occupancy.”

6) Since this PLOS Biol. and not PLOS computational biol. I wonder if there are certain descriptions that may be written in less technical language. I realize that this cannot be done for the entire text and so one might argue that the technical level is what it is and the reader will have to manage. Using terms like Principal Component Analysis in the text right beside the more technical description of "performing an eigenvalue decomposition of the covariance matrix" may give less advanced readers "a hook" to pursue a reference to help them with their understanding.
We thank the reviewer for the suggestion. In the revised manuscript where possible we have tried to introduce a technical term using less technical language which should help readers that are not experts in the behavior field to get a better understanding of what we are trying to achieve by employing a particular analysis technique . Examples of the additional text we included in the revised manuscripted (underlined here) include but are not limited to: 1. ‘We confirmed this by performing Principal Components Analysis in the form of an eigenvalue decomposition of the covariance matrix’ ; 2. ‘First, we used a summary statistic called Standardized Median Difference’. 3. ‘we decided to use matrix profile, a computational tool that makes it possible to solve the dual problem of motif discovery and anomaly detection in a time series dataset’. 4. ‘simple Gaussian Hidden Markov model (G-HMM), which is a state-based statistical model’)

Minor comments:

In general I found the figures to be good, but there are many readability issues throughout. In many cases the axis labels are inconsistent in size and need to be enlarged. For example, Fig 1b has readable x-axis labels (EC1, EC2, etc) but the y-axis labels (-0.2, -0.1, etc) are very small even though there is plenty of space to increase the size. This happens throughout where the label sizes are mixed and often one axis label is very difficult to read.
We thank the reviewer for pointing out this issue and we have done our best to address it in the revised manuscript. We have increased the font sizes of labels not only in Figure 1 but across all main and supplemental figures. Where possible we have tried to increase font size to Arial 11pt or greater. In some cases, we have split the figures (e.g. old S1 and S2 Figs are now split to four figures: S3-S6 Figures). We hope that the reviewer will find that the readability of the labels has improved.

The following are very minor typos or inconsistent references to chemicals. Instead of trying to map these out in the text, I think these can be found by the authors using the search function.

"Segments speeds'" should be "segments' speeds"

Corrected
"Rapid holt" — "Rapid halt"?
Corrected
Fig 3 F and Fig 3F (inconsistent figure referencing)
Corrected to Fig 3F.
"wild type animal" - "wild-type animal"
Corrected throughout the manuscript.
Berman et al[7] - Berman et al.[7]
Corrected
"eigen coefficient features" - "eigen-coefficient features"
Corrected
"Mammping" - "mapping"
Corrected
Chemicals are sometimes written as "ascorbic acid" and "Ascorbic Acid"
Corrected throughout the text to Ascorbic acid.
"python" maybe should be capitalized.
Corrected
I scanned the supplemental information. It is not clear to me that all the movies are important to show beyond the plotted supplementary figure.

We thank the reviewer for this remark. We agree that there are numerous videos, which are indeed summarized in the supplemental figures. In the revised supplemental information, we include the animation of a larva exploring the tSNE behavioral space and the two examples of startle-like behavior. All other videos have been removed.


Reviewer #2:

"Comprehensive analysis of locomotion dynamics in Ciona intestinalis reveals how neuromodulators flexibly shape the behavioural repertoire of a protochordate" by Athira et al. takes a series of computational ethology methods applied to other organisms and applies them to Ciona intestinalis larvae.  This is worthwhile because, as the authors argue, Ciona has a compact nervous system and is a close relative of vertebrates, putting it at a unique niche with respect to evolution and neural complexity. At the same time, its behaviour has not yet been analysed using the latest computational tools.  The quantification and visualisation of its behavioural repertoire and its modulation by light and drugs is therefore a useful contribution.
We thank the reviewer for their kind comments. We were largely inspired by and leveraged computational ethology methods and open-source tools (such as Tierpsy) used in established model organisms like C. elegans, Drosophila and mouse. We hope that our manuscript will further incentivise neurobiologists working with non-model organisms, including tunicates to perform behavioral studies using modern computational tools.

The paper is mostly clear, but I think it has two main weaknesses.  The first is that three different methods are applied to the data but there is very little discussion of how the results relate to each other.  Are there any advantages to one approach over another?  Are they complementary or orthogonal (or something else) in their outputs?
We thank the reviewer for the suggestion to compare the three different methods and how the results relate to each other. In the revised manuscript’s Discussion section, we have added a new subsection titled “Comparison of complementary computational ethology methods used to quantify the behavioral repertoire of Ciona.”. In this subsection we attempt to highlight the advantages and disadvantages of each approach and discuss how the results coming out of each method relate to each other.
The second major weakness of the work is that the results of the different quantification methods are difficult to interpret without statistical tests or some other measure of their informativeness. For example, there are many claims about a drug affecting some aspect of one of the quantification methods, but no indication of whether the result is due to a drug effect, noise, a batch effect, or something else.  Specific cases are listed below.
We thank the reviewer for bringing up this important point. We have performed statistical analysis for the results of the different quantification methods. In the cases where our results/claims were backed-up by the statistical analysis we have kept our statements, however in the cases that our claims were not supported following the statistical analyses we have removed them from the manuscript. Please see our responses to the specific cases listed below. Overall, we are grateful to the reviewer because their suggestion has been important for improving the manuscript’s quality and the robustness of our conclusions.

Finally, an issue in multiple places, again listed below, is the presence of unsubstantiated claims that are not supported by the data.  I believe these can be mostly addressed with re-writing, but together they may substantially change the paper, including the second half of the abstract.
We thank the reviewer for raising these points. We have tried our best to address these either by adding new data or by re-writing. As a general trend in the revised manuscript we have tried to be more cautious and restrained in our claims. Please see our responses below.

Specific points:
-"which are likely left undetected with less sensitive methodologies". Without being specific or comparing to other methods, this claim should be removed.
We thank the reviewer for the suggestion. This claim has been removed.

-"Curvature is defined as inversely proportional to the radius of the osculating circle at a given point on the skeleton". The circles in the schematic figure seem to be oriented the wrong way, i.e. showing the opposite curvature to the skeleton.
We apologise for the mistake in the schematic. We now provide a revised panel F in Figure 1 where we have corrected the orientation of the osculating circles.

-"software[38]" This software was first published in Javer et al. (2018) Nature Methods.
We apologise for the omission. We have added Javer et al. 2018, Nature Methods reference.

-"a scalar measure of eccentricity".  Eccentricity is a scalar.  Perhaps a related measure is better.
Thank you for the suggestion, we have corrected this to read ‘a related measure of eccentricity’.

-When calculating the eigencionas in the results, it would make sense to cite the original eigenworms paper.  I realise it is cited elsewhere already, but I would cite it here as well.  The same goes for the results sections on motifs and the HMM.

We thank the reviewer for the suggestion. We have added Stephens et al PlosCompBio 2008 (2) to all the relevant Results subsections. In the subsection titled: “Parametrization of Ciona using interpretable features” it has been added in two spots: “…without losing significant information…”

and “…eigenvalue decomposition of the covariance matrix…”. The reviewer’s comment prompted us to cite Stephens et al as well as other methods papers for each of the analysis techniques used. It has also been added to the analytical approaches that we employed which make use of the eigencoefficient features, i.e. for HMM analysis after the phrase “any of these states, take a range of eigencoefficient features” and for Spatio-temporal embedding after the phrase “…we used as the input the 6 eigencoefficient features…”.

This comment of the reviewer also prompted us to try to make sure we cite original and/or key review articles for all the analytical approaches we have used. These include the following: In the Results subsection: “Ciona locomotion is rich in behavioral motifs across timescales.” we cite Brown et al PNAS 2013(3) and Fu Eng. Appl. Artif. Intell., 2011(4). For the subsection: Ciona motor behavior can be modelled in terms of states and transitions.” we have added following the phrase “Gaussian Hidden Markov model (G-HMM), which is a state-based statistical model” Rabiner, Proc. IEEE,1989(5).

-"Like EC1 and in contrast to EC2, EC3 exhibits a very strong positive trend across most drug treatments."  Which of these changes are significant?
We thank the reviewer for the question. We have now included table S48 in the supplementary information document, which summarizes in a reader friendly manner the drugs that exhibited statistically significant differences for each EC (detailed statistics are provided in S11-S13 Tables). Fig. S6 includes violin plots showing the distribution of the six ECs for different drugs in comparison to the respective control. In all panels drugs which showed significantly higher values of the feature are grouped together within a red-colored border whereas drugs with significantly lower feature values with respect to controls were grouped within a blue border.

While we discuss some of the drugs that result in significantly different EC values in the main results text, we opted for a summary table since exhaustively listing all the significantly different changes in the main text might be tiring for the reader. In the revised manuscript we encourage the reader to look at the supplemental table with the following sentence included in the main text: “Drug treatments that resulted in statistically significant changes in EC values are summarized in Table S48.”

-"recurring motives and anomalies in the data" -> "recurring motifs and anomalies in the data"
We thank the reviewer for pointing out this error. We have modified the text in this paragraph and corrected the error as well.

-Figure is too big/labels too small.  Even in the large tiff version of the figure, most detail is lost in the time series plots and the labels are difficult to read.
The reviewer rightly points out this issue or readability of the labels. The same issue was also brought up by reviewer #1. We have addressed the issue by increasing font size of all labels across all figures (main and supplemental).

-"inertia of the model considerably less".  I understand the idea but I think this could be rephrased to be more specific.
We thank the reviewer for the suggestion. We have replaced the pharse highlighted by the reviewer with: “using the Elbow method, where we find the maximum of the second derivative of the curve showing variance explained over cluster numbers.”.

-"Raclopride treatment results in a strong reduction in the representation of Clusters "A1" to "D1"". for this and all of the related claims, which changes are significant?  By eye, it looks like the effect of Raclopride might be the only significant one.
We thank the reviewer for raising this important point. We have now performed statistical analysis on the effects of the drugs on the usage of the clusters in the Motif, HMM and spatiotemporal embedding analyses. Detailed information on p-values, sample size effect, etc for each analysis approach can be found in Tables S36-S47 (excel file) and the results are summarized in a reader friendly manner in Tables S49 to S52 in the Supplementary Information word document. We find that several our claims with regards to the effects of the drug treatments on cluster usage still hold after statistical analyses, however some of the claims are not supported anymore by the statistics and therefore we have modified the text accordingly by removing these claims. With regards to the example that the reviewer mentioned we find that Motif Cluster A1 is indeed significantly reduced by Raclopride by this is not the case for B1 to D1 clusters as previously claimed in the text. Thus, this claim (with regards to B1 to D1 clusters) has been removed. Details regarding the additional statistical test methods performed to address the question of the reviewer are included in the last paragraph of the Methods subsection titled: Statistics.

-" indicating that the behavioural motifs included in these clusters may be generated by a common underlying cellular and/or molecular mechanism." It's also consistent (and seems more likely) that they are simply similar motifs or are part of a larger motif so that they often occur together.
We thank the reviewer for the suggestion. In the revised manuscript the text reads as follows: “Our findings could be explained in multiple ways. The more likely possibility is that the two clusters contain similar motifs or that they are parts of a larger motif thus they often occur together. Another possibility is that they are generated by a common underlying cellular and/or molecular mechanism.’

-"underlying states consistently across experiments and datasets." Rather than consistency across experiments, what follows seems to be a manual annotation.  It might be more useful to quantify how similar the states are when the data are resampled or when different experiments are analysed independently.
We thank the reviewer for this comment. We have now modified the sentence to read: “underlying states across experiments and datasets.” Nonetheless, we have now trained multiple additional 10-state Hidden Markov models to study the similarity and differences in inferences about the structure of behaviour when presented with similar or different data. All the supporting plots are provided in supplemental figures Fig. S16. and Fig. S17. As a first step, we fit 2 new 10-state HMM models with the entire dataset (1743 videos as we did for the original model) and examined the state transition probability matrices, Gaussian emission probabilities and skeletons sampled from each state for the original and the 2 new models. Although the states do not have a one-to-one correspondence, we could infer some similarity from these comparisons. For example, state η in the original model and state 5 in the new model 2 and state 6 in the new model 3 exhibit similar trends in transition probabilities (Fig S16 panels J, K and L respectively). In addition, we also looked at the means and standard deviations of the emission probabilities of these states (Fig S.16. panels A, B and C) and a similar trend is seen there as well. These trends are also seen in the sampled skeletons from these three states (state η in the original model, Fig S17 panel A showing skeletons from new model 2 and Fig S17 panel B showing skeletons from in the new model 3) suggesting that they all correspond to an actively exploring state. Similar correspondences can be inferred for state β in the original model, state 2 in the new model 2 and state 3 in the new model 3. Additionally, we trained 3 10-state HMMs with mutually exclusive subsets of the original dataset, being agnostic to if the data corresponds to animals subjected to any drug treatment or light stimuli. Similarly, we trained another 3 10-state HMMs, again with mutually exclusive subsets of the original dataset, although in this case we sampled the videos such that the same number of control, drug and light experiments are the same across each of the 3 datasets. The results from the 3 models trained with these randomly split subsets and the 3 models trained with uniformly split subsets indicate that the inferences made by models differ slightly in terms of the transition probabilities when trained with different data (supplemental figure Fig S.16). However, as seen from the skeletons sampled from the learned states and the means and standard deviations, the overall ratio of high postural variance (indicating active exploration) to very low postural variance states remain more or less the same (supplemental figure Fig S.17).

-"we demonstrate that in Fluoxetine, states "α" and "κ" are overrepresented in comparison to the wild type".  Are these differences significant?
We thank the reviewer for raising this question. We have performed statistical analysis for all the HMM state representation comparisons between wild-type and drug treated animals. The stats results for the HMM analysis can be found in Tables S42-S44 and summarized in a reader friendly manner in Table S51. With regards to the specific difference (i.e. the effect of Fluoxetine on states "α" and "κ") this statement holds true after statistical analysis. However, some other differences that we discussed in the original manuscript were found not to be statistically significant, therefore we have removed these claims from the results and discussion section.

-"Our results show that the clusters identified by this method are coherent across datasets. For example, cluster 1 consistently represents video frames where the larva is actively swimming and exploring the arena".  As before, this doesn't really demonstrate consistency of the state.  If one were to simply define a state as all cases where the larvae are slower than a threshold, then confirming that the larvae in that state are indeed slow doesn't provide evidence that the state is meaningful.
We thank the reviewer for pointing out this. We would like to apologize for not being clear enough about how we define the clusters. The input feature set that is used to define the behavioural space is strictly the eigen coefficient features. Once we find this lower dimensional projection, we then cluster the projections using automated DBSCAN algorithm. When we randomly sampled *Ciona* skeletons from each of these clusters and visually examined them, we could observe that the clusters exhibit some coherence in terms of variance in postures. Visual examination of the trajectories in the arena also suggests a similar trend, for example, frames classified as cluster 1 seems to correspond to the frames where the larvae is actively exploring the arena. We have included an animation (supplementary data movie S3) of an animal exploring the behavioural space simultaneously showing its trajectory in the arena and the speed values is 3 body segments. Although the speed features are not used in this analysis, we observe that the speed values are higher when the animal is in the cluster 1 and the trajectory also suggests an active exploration in the arena.

-"is more comprehensive than the discrete biophysical features such as segment speeds, curvatures and tangent angles; classically important parameters for describing motor behaviour."  This may be true in some sense, but it needs to be made specific and supported by data.  I would also drop "remarkably" from the start of the next sentence given the previous work cited in the sentence after that.
We thank the reviewer for this comment. Given that we choose the eigencionas based on the fact that they explain 97% of the variance in the 49-point curvature data, we then tried to compute the percentage of variance accounted by the segment curvatures. The 7 segment curvatures together explain only 28% of the variance observed in the 49-point curvature data. This suggests that when compared to choosing 7 segments in the way described in the paper, eigencionas gives a lower dimensional representation of the data with lesser loss of information. However, we have decided to modify the sentence that the reviewer is referring to read as follows: “Through this approach, we measured biophysical features such as segment speeds, curvatures and tangent angles; classically important parameters for describing motor behavior which were not available to us when we used centroid tracking”.

In addition, we have dropped “remarkably” from the start of the sentence.

-"reveal its modular structure".  It's more accurate to say that the methods impose a modular structure on the behaviour.

We thank the reviewer for the comment. We have modified the text and now it reads as follows: ‘use multiple complementary approaches that impose a modular structure on the behavioral dynamics of *Ciona*.’

-"Model fitting preserves temporal component to expose biologically meaningful motor modules and transitions". Without confirming that the changes in module use are significant, it's difficult to argue they are biologically meaningful.

We thank the reviewer for the suggestion. While we performed statistical analysis on the changes in module use and we show that some are indeed statistically significant (please see S42-S44 and S51 Tables), we would rather be cautions on our interpretation and thus in the revised manuscript the subsection title reads: ‘Model fitting preserves temporal component to expose motor modules and transitions’.

-"we have shown that Ciona larval locomotion can be decomposed into 10 distinct stereotyped locomotor states".  I would rephrase this, since the statement would still be true for any number of states.
We have rephrased this to read ‘multiple distinct stereotyped locomotor states’

-"This richness of locomotor states comes as a surprise, since they are the output of a relatively simple nervous system equipped with a minimal number of neurons that make up the motor circuit of the larva[21, 22]".  Again, given the previous applications of these methods to other systems, I would remove the reference to surprise. 
We have removed the reference to surprise and the revised text reads as follows: “Despite a relatively simple nervous system equipped with a minimal number of neurons that make up the motor circuit Ciona larvae exhibit multiple locomotor states.”

-"Our findings show that both serotonin and dopamine regulate the transition statistics between the beating and gliding states, pointing to a more complicated modulation of the beat-and-glide behaviour across species compared to what was previously thought."  Possibly, but could be off-target effects of the compounds.  Also, the null model that an important neurotransmitter has no effect on, e.g. beat-and-glide, seems unlikely to start with.  More detail of what the 'previous expectations' are and whether these results contradict them would be required to support this statement.
We thank the reviewer for raising this point and we apologise for the lack of clarity from our side. Our intention was to point out that in zebrafish beat-and-glide behavior has been shown to be under the control of dopamine even though not all parameters were influenced as a result of perturbations of dopamine signalling(6). To the best of our knowledge serotonin has not been implicated in the beat-and-glide behavior in zebrafish, however we agree with the reviewer that there is no reason to anticipate that this important neuromodulator would have no effect on the behavior in fish or marine invertebrates. It may very well be that this is just due to a lack of studies investigating the role of serotonin in the beat-and-glide behavior in zebrafish.

Furthermore, what we would like to achieve is to encourage others in the community to look for and possibly identifying similar behaviors across organisms occupying key positions in the chordate phylogeny. Subsequently by looking at the signalling pathways that control these behaviors across species we may be able to generate some hypotheses regarding the evolution of chordate nervous systems and behaviors.

In any case, we have removed that sentence and replaced it with the following: “Our findings indicate that both serotonin and dopamine are important for executing this behavior. From an evolutionary perspective it would be interesting to determine whether other invertebrate chordates such as amphioxus and Oikopleura doica are able to perform a beat-and-glide like behavior and to which extent this behavior may be under the control of the same or different neuromodulators across chordates.”

-"states appear to be selectively modulated by bioamines."  This statement is also not supported by the data.  Selectively would imply that these changes are seen when levels of bioamines are changed but not other perturbations.  In fact, given that the model output is basically time spent in states and the transition probabilities between them, anything that effects their behaviour would change one or both of these.
We thank the reviewer for the comment. We agree that our screen is far from exhaustive in terms of perturbations, so we cannot categorically use the term ‘selectively’, thus we have removed it from the sentence.

-"That, the key clusters and transitions involved in the behaviour were preserved under all pharmacological treatments suggests that these features are controlled by neuromodulators other than bioamines e.g. GABA and Glutamate. However, the transition statistics were demonstrated to be affected by dopamine and serotonin (which is in line with the HMM findings), indicating that bioamines likely perform the fine sculpting of this behaviour,"  It is also possible that these drugs do not accumulate in the larvae at a high enough concentration to act or that their targets are not sufficiently conserved to have the expected effect.  The discussion of fine sculpting vs some other kind of change should be clarified or removed.

We have removed this discussion section since the statements made are largely speculative and as the reviewer rightly points out the absence of an effect in some cases could be attributed to experimental limitations with regards to the efficacy of the pharmacological perturbations.

-"Here we have shown that Ciona larvae are able to use a finite number of behavioural modules".  This isn't a result so much as a property of the analysis method.
We thank the reviewer for the comment. We have replaced this text it with the following sentence: “As a result of the multiple analytical methods we have employed in our study we have broken down relatively complex larval behaviors to simpler modules …”. We hope that this sentence is in line with the reviewer’s suggestion.
-"here that bioamines act as behavioural switches" This should be made precise and supported by data or removed.
We have removed this sentence.

-"our findings indicate that these behavioural modules represent a class of druggable targets".  I would rephrase this.  Druggable targets would normally refer to a protein target not a behaviour.
We have removed this statement from the end of the paragraph and replaced it with a sentence discussing how in the future we can leverage our behavioral analysis approach in combination with functional imaging and genetic mutants to systematically dissect the role of bioamines in modulating neuronal activity and behavior in Ciona.


Reviewer #3:
[identifies himself as Takehiro G. Kusakabe]

The authors' group has been using the Ciona intestinalis larva as a model animal for neurobiology and behavioral study. In this manuscript, the authors extended their previous studies to computational modeling of swimming dynamics and applied the method to pharmacological evaluation of bioamines effects and to analyze behavioral response to photic stimuli. The authors conducted high-throughput analysis of a large number of larvae and revealed that postural variance of Ciona larvae can be classified into 6 basic shapes, which they call "Engenciona". Then, the authors computationally analyzed temporal patterns of posture series, and compared the patterns between control larvae with experimental larvae (drug treated and different light stimuli). Their findings suggests roles of bioamines in specific neural and behavioral processes. The authors also propose possible conservation between Ciona larval behavior and vertebrate behavior. This study is highly original and provides a novel platform for studying neural and behavioral mechanisms of this emerging model organism. Therefore, this work potentially has a substantial impact on various biological fields, including neurobiology, physiology and ethology. However, there are some problems which should be solved before its acceptance for publication in PLoS Biology.

My major concerns are insufficient descriptions of experimental methods and unclear explanation of relationships between this study and a previous work by the same group (Rudolf et al. Sci. Rep. 2019).
We would like to thank Professor Kusakabe for his kind comments and we have done our best to address his concerns in the revised manuscript.

1. The authors only provide "Drug concentrations Table" to explain their pharmacological assays. They should provide detail information for each chemicals, such as manufacturers, product codes, and solvents (DMSO, ethanol, etc.).

We thank Prof. Kusakabe for pointing out this omission. In the revised Drugs concentrations Table, we include the manufacturers, the product codes. In the newly added methods subsection titled: “Experimental procedure” we know clarify that all drugs were dissolved in ASW (Artificial Sea Water) except for Dopamine which was dissolved in ASW+ Ascorbic Acid (28 μM) to prevent oxidation of Dopamine.

2. It is difficult to understand detailed design and use of their experimental setup (Ciona Tracker 2.0) for behavior recordings from a short sentence on the first page of Methods and relatively low resolution images presented in Fig. 1A. For example, what type of container did they use to keep the larva during recording? Petri dish or custom-made container? What size? Amount of medium (sea water?)? Any lid?? What temperature? Light source (infrared?) for visualization?
We thank Prof. Kusakabe for pointing out these issues. Some of these were also raised by reviewer # 1 so we have done our best to address them. We have now included a detailed schematic of Ciona Tracker 2.0 in Figure 1A and a high-resolution picture of the animal in the arena in Figure 1B. We have also a new supplementary Figure (Fig. S1) with photographs of the process of generating arenas, as well as the different parts of the setup (including photographs and 3D renderings of certain parts).

Importantly, to provide more information on the setups and experimental procedure we have substantially extended the following methods subsections: “Experimental Setup” and “Experimental procedure”. Amongst many details that we now provide in these sections we have made sure to explain how we prepare the agarose-based arenas, we provide their dimensions and the amount of ASW that each arena can hold. We provide details on how we generate the dark field illumination with IR LEDs and we discuss how we monitor and maintain the temperature at 14°C.

3. This group previously reported a paper of similar subject (Rudolf et al. Sci. Rep. 2019). The present manuscript refers to this previous paper (ref. 52), but it is unclear what was previously found or achieved and what are new in the present study. These points should be explained.
We thank Prof. Kusakabe for raising this point. This was indeed an important omission. We have now added multiple sections in the discussion outlining some key technical and biological advances of this work relative to the Rudolf et al. paper.

In the revised Discussion section under the subsection titled: “Motion tracking and comprehensive feature extraction at high-throughput, resolution and reliability” we discuss the benefits of using midline tracking (this manuscript) vs centroid tracking (Rudolf et al), in terms of being able to capture larval body posture, which was used to detect behaviors that are not detectable by centroid data and to reveal subtle phenotypes resulting from perturbations (pharmacological in this case) using features such as curvature.

In addition, in the revised discussion subsections titled: “Motif analysis enables generation of a multiscale dynamic behavioral representation” and “Model fitting preserves temporal component to expose motor modules and transitions” we compare the simple ontology of behavioral modes of swimming we obtained in Rudolf et al using agglomerative clustering, to the results of the motif and HMM analysis approaches we used in this study. We discuss the greater richness of behaviors that we detect in this manuscript (Athira et al), as well the opportunity we have in this study relative to Rudolf et al to study the transitions between behavioral modes. Finally, in the discussion subsection “Summary and Outlook” we discuss how in Athira et al we were able to report on the contributions of multiple bioamines on behavioral dynamics due to the higher throughput and higher resolution that our new approach enabled us.

Minor points:
4. It is unclear the merit of naming "Eigenciona" for basic postural patterns. The authors use various inconsistent expressions, such as eigenvectors, eigen vectors, eigen features, Eigenciona vectors, Eigenciona, eigen Cionas, eigen-cionas, etc.
The use of the term ‘eigencionas’ term was inspired by multiple papers across different organisms that use the (eigenspecies) term to refer to eigenvectors specific to the species studied (e.g. worms(2), Drosophila maggots(7) and zebrafish(8)). This is briefly discussed in the discussion subsection “Motion tracking and comprehensive feature extraction at high-throughput, resolution and reliability”.

We apologise for the inconsistent use of eigen based expressions. In the revised manuscript we have made a strong effort to be more consistent. In the main text of the revised manuscript, we primarily use the terms ‘eigencionas’ and ‘eigencoefficients’. When we first bring up eigenvectors in the revised manuscript, we explain that from that point onwards we will use eigencionas to mean eigenvectors. Since eigencoefficient refers to something distinct from eigencionas/eigenvectors we have also kept this term. However, all other variants of eigen have been largely eliminated.

5. Last paragraph of Introduction, 7th line: lavae --> larvae

We thank Prof. Kusakabe for pointing this spelling mistake out. It is now corrected.

6. In the paragraph after the legend for Fig. 2: two "for EC2" are contained in a sentence. It may be redundant.

Thank you for spotting this. We removed the 2^nd^ "for EC2".

1.	Nakagawa M, Miyamoto T, Ohkuma M, Tsuda M. Action Spectrum for the Photophobic Response of Ciona intestinalis (Ascidieacea, Urochordata) Larvae Implicates Retinal Protein. Photochemistry and Photobiology. 1999;70(3):359-62.

2.	Stephens GJ, Johnson-Kerner B, Bialek W, Ryu WS. Dimensionality and dynamics in the behavior of C. elegans. PLoS Comput Biol. 2008;4(4):e1000028.

3.	Brown AE, Yemini EI, Grundy LJ, Jucikas T, Schafer WR. A dictionary of behavioral motifs reveals clusters of genes affecting Caenorhabditis elegans locomotion. Proc Natl Acad Sci U S A. 2013;110(2):791-6.

4.	Fu T-c. A review on time series data mining. Engineering Applications of Artificial Intelligence. 2011;24(1):164-81.

5.	Rabiner LR. A tutorial on hidden Markov models and selected applications in speech recognition. Proceedings of the IEEE. 1989;77(2):257-86.

6.	Jay M, De Faveri F, McDearmid JR. Firing dynamics and modulatory actions of supraspinal dopaminergic neurons during zebrafish locomotor behavior. Curr Biol. 2015;25(4):435-44.

7.	Szigeti B, Deogade A, Webb B. Searching for motifs in the behaviour of larval Drosophila melanogaster and Caenorhabditis elegans reveals continuity between behavioural states. J R Soc Interface. 2015;12(113):20150899.

8.	Mearns DS, Donovan JC, Fernandes AM, Semmelhack JL, Baier H. Deconstructing Hunting Behavior Reveals a Tightly Coupled Stimulus-Response Loop. Curr Biol. 2020;30(1):54-69 e9.
